# *Dictyostelium *transcriptional responses to *Pseudomonas aeruginosa*: common and specific effects from PAO1 and PA14 strains

**DOI:** 10.1186/1471-2180-8-109

**Published:** 2008-06-30

**Authors:** Sergio Carilla-Latorre, Javier Calvo-Garrido, Gareth Bloomfield, Jason Skelton, Robert R Kay, Alasdair Ivens, José L Martinez, Ricardo Escalante

**Affiliations:** 1 Instituto de Investigaciones Biomédicas Alberto Sols, Universidad Autónoma de Madrid-Consejo Superior de Investigaciones Científicas, Madrid, Spain; 2 MRC Laboratory of Molecular Biology, Cambridge, UK; 3 Wellcome Trust Sanger Institute, Hinxton, UK; 4 Centro Nacional de Biotecnología, CSIC, Madrid and CIBERESP, Spain

## Abstract

**Background:**

*Pseudomonas aeruginosa *is one of the most relevant human opportunistic bacterial pathogens. Two strains (PAO1 and PA14) have been mainly used as models for studying virulence of *P. aeruginosa*. The strain PA14 is more virulent than PAO1 in a wide range of hosts including insects, nematodes and plants. Whereas some of the differences might be attributable to concerted action of determinants encoded in pathogenicity islands present in the genome of PA14, a global analysis of the differential host responses to these *P. aeruginosa *strains has not been addressed. Little is known about the host response to infection with *P. aeruginosa *and whether or not the global host transcription is being affected as a defense mechanism or altered in the benefit of the pathogen. Since the social amoeba *Dictyostelium discoideum *is a suitable host to study virulence of *P. aeruginosa *and other pathogens, we used available genomic tools in this model system to study the transcriptional host response to *P. aeruginosa *infection.

**Results:**

We have compared the virulence of the *P. aeruginosa *PAO1 and PA14 using *D. discoideum *and studied the transcriptional response of the amoeba upon infection. Our results showed that PA14 is more virulent in *Dictyostelium *than PA01using different plating assays. For studying the differential response of the host to infection by these model strains, *D. discoideum *cells were exposed to either *P. aeruginosa *PAO1 or *P. aeruginosa *PA14 (mixed with an excess of the non-pathogenic bacterium *Klebsiella aerogenes *as food supply) and after 4 hours, cellular RNA extracted. A three-way comparison was made using whole-genome *D. discoideum *microarrays between RNA samples from cells treated with the two different strains and control cells exposed only to *K. aerogenes*. The transcriptomic analyses have shown the existence of common and specific responses to infection. The expression of 364 genes changed in a similar way upon infection with one or another strain, whereas 169 genes were differentially regulated depending on whether the infecting strain was either *P. aeruginosa *PAO1 or PA14. Effects on metabolism, signalling, stress response and cell cycle can be inferred from the genes affected.

**Conclusion:**

Our results show that pathogenic *Pseudomonas *strains invoke both a common transcriptional response from *Dictyostelium *and a strain specific one, indicating that the infective process of bacterial pathogens can be strain-specific and is more complex than previously thought.

## Background

Nosocomial infections caused by opportunistic pathogens are one of the most important health problems in developed countries. Depending on the geographic location, *P. aeruginosa *is the first or second causative agent of nosocomial infections [1,2]. *P. aeruginosa *infects patients suffering from AIDS, people at intensive care units, and burned people among others, and is the major cause of morbidity and mortality in patients with cystic fibrosis, the most prevalent hereditary disease in Caucasian populations [3]. A successful infection by this type of pathogens depends on the interplay of multiple factors including the susceptibility of the host, the virulence of the strain and its resistance to antibiotics [4]. Previous work has shown that the physiological fitness and the virulence of *P. aeruginosa *and other opportunists are affected by the expression of antibiotic resistance mechanisms such as MDR-pumping systems [5-8].

The pathogenicity of *Pseudomonas aeruginosa *involves various components operating at different levels. The flagella and *pili *facilitate contact with the bacterium's cell target and play a role in its adhesion, which is a critical step in the infection [9,10]. After contact, the type III secretion system is able to inject into the cytoplasm of the target cell a series of cytotoxic molecules that act at various levels. The mechanism of action involves, in many cases, the presence of host cofactors still unidentified [11]. Other virulence factors involve products secreted into the extracellular medium by systems I and II such as elastase, alkaline phosphatase and exotoxin A among others. The expression of many of these virulence factors is regulated by a mechanism of bacteria-to-bacteria cell signalling known as quorum-sensing [12]. Despite the functional and genomic similarity among different *P. aeruginosa *strains [13,14], some differences in their pathogenicity have been observed [15]. For example, the clinical isolate PA14 is more virulent than PAO1 in a wide range of hosts [15-17]. It has been shown that the genome of PA14 contains two pathogenicity islands that are not present in PAO1 and it has been proposed that the virulence in this organism (and the difference between PA14 and PAO1) is the result of a pool of pathogenicity genes interacting in various combinations in different genetic backgrounds [15]. In spite of these suggestions, the cause of the different virulence behavior of PAO1 and PA14 is not yet fully understood.

Although most of the work on pathogenesis has been focused on understanding the bacterial factors that render a virulence phenotype, increasing attention is being paid to the host and those aspects connected to the susceptibility or resistance to infection. Understanding the host-pathogen relationship, at both the cellular and molecular level, is essential to identify new targets and develop new strategies to fight infection. Molecular analysis of host-pathogen interactions would benefit from the use of model systems allowing a systematic study of the factors involved. In this regard the social amoeba *D. discoideum *has proven particularly useful for its ease of handling, genetic tractability [18-22] and fully sequenced genome [23].

*D. discoideum *is a soil microorganism that feeds on bacteria by phagocytosis. The interaction between bacteria and their natural predators (*Dictyostelium*, other protists and worms) is believed to have shaped both predators [24] and bacterial evolution. As a consequence, some of the mechanisms developed by bacteria to avoid the activity of their natural predators in the environment might have been adapted later in evolution to allow the infection of higher organisms such as humans [25]. Specifically, it was found that the quorum-sensing mechanisms and type III secretion, which are essential factors in the infectivity to humans are also responsible for the infectivity of *P. aeruginosa *in *D. discoideum *[18,20,21].

Our previous studies have shown the utility of this model system of infection to analyze the virulence of other opportunistic pathogens like *Stenotrophomonas maltophilia *[7]. It has been also demonstrated the validity of *D. discoideum *as a model of infection by intracellular pathogens such as *Legionella, Cryptococcus and Mycobacterium *[19,22]. Consequently, the conservation of the mechanisms of infection needed to infect mammals and *D. discoideum *in a wide variety of pathogens reinforces the use of this system as a valid model to study host-pathogen relations. We have used whole-genome *D. discoideum *microarrays to study global host transcription upon infection with *Pseudomonas aeruginosa *PAO1 and PA14 to determine whether or not transcription is being affected as a defense mechanism or altered in the benefit of the pathogen.

## Results

### *Pseudomonas aeruginosa *strains PAO1 and PA14 show a different virulence behavior in *D. discoideum*

**Figure 1:**
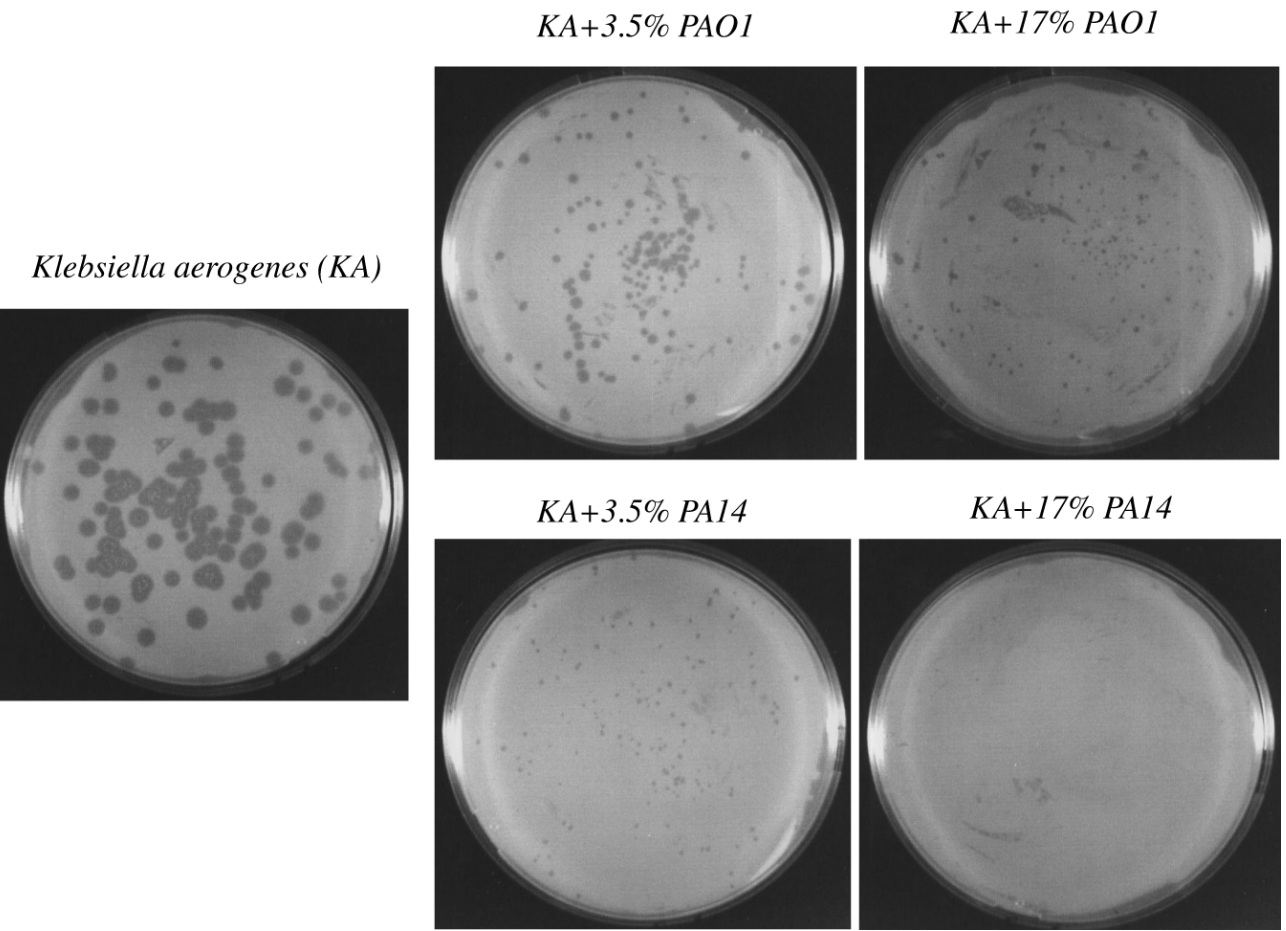
**PA14 is more virulent than PAO1 in SM-plating assay**. Approximately 100 *D. discoideum *cells were cultivated in SM-plates with the indicated proportion of *Klebsiella *and *Pseudomonas *strains (PAO1 or PA14) previously grown and adjusted to the same optical density. Plates were maintained at 22°C for 5 days. Growth of *D. discoideum *is severely affected by the presence of *Pseudomonas *but the inhibition is stronger when PA14 is used.

**Figure 2:**
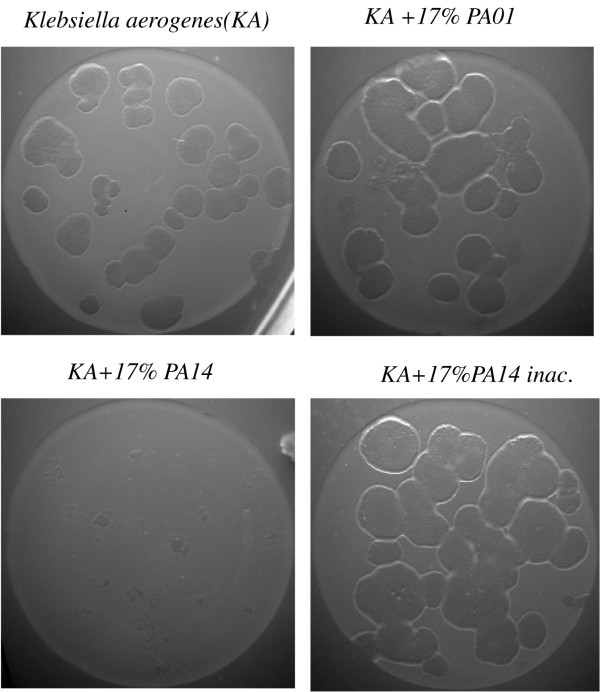
**PA14 is more virulent than PAO1 in PDF-agar plating assay**. *D. discoideum *cells were cultivated in non-nutrient agar on a lawn of *Klebsiella *and *Pseudomonas *(PAO1 and PA14) at the indicated proportion. Under these conditions PA14 maintain a high virulence as seen by the strong inhibition of *D. discoideum *growth. Heat inactivated PA14 is used as a control.

PAO1 and PA14 are two clinical isolates of *P. aeruginosa *frequently used as model strains to analyze the virulence of this bacterial pathogen. Since they behave differently in some aspects dealing with the expression of virulence determinants, we wanted to compare the differential response of the host to these strains. For this purpose, we made use of *D. discoideum *as a model for virulence. As a first step a plating assay of virulence was set up. Figure 1 shows a representative experiment of three independent assays in which *D. discoideum *cells were grown in association with bacteria on nutrient SM plates. *Klebsiella aerogenes*, a non -pathogenic bacteria, was used as an appropriate food supply and *P. aeruginosa *mixed at the indicated proportions. An effect in the size of the clearing plaques could already be seen when only 3.5% of *P. aeruginosa *cells were mixed with 96.5% of *K. aerogenes *cells and this effect was even clearer using 17% of *P. aeruginosa *cells. When the behavior of the strains was analyzed in more detail, it was found that PAO1 is reproducibly more permissive than PA14 as observed by the higher growth of *D. discoideum *on PAO1. The differences in the area of the cleared bacterial lawn between PAO1 and PA14 were measured for the condition corresponding to the 3.5 % mixture. The average area and the standard deviation were 1.65 ± 1.2 mm^2 ^for PAO1 and 0.11 ± 0.07 mm^2 ^for PA14 (the number of clear plaques measured in each condition was 50). The significance of differences between groups as determined by Student's *t*-test was p < 10^-8^. To further confirm these results a different plating assay was performed on non-nutrient agar. PAO1, PA14 and *K. aerogenes *were previously grown in LB overnight, washed out of the media by centrifugation and deposited with *D. discoideum *cells in agar plates at the indicated proportions. Under these conditions the difference in the virulence between PAO1 and PA14 was even more evident as shown in a representative experiment in Figure 2. Interestingly PAO1 is permissive to *D. discoideum *growth under these non-nutrient conditions. However, PA14 still shows a strong virulence against *D. discoideum*. All together these results suggest that PA14 is more virulent than PAO1 in the *D. discoideum *model of virulence.

### *Pseudomonas aeruginosa *induces a specific gene expression response in *Dictyostelium*

**Figure 3:**
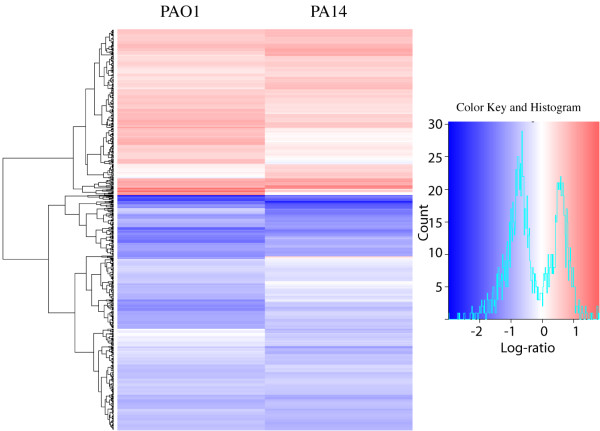
***Pseudomonas aeruginosa *induces gene expression changes in *Dictyostelium***. Heat map comparing the genes significantly altered (p < 0.05) between PAO1-treated cells versus control (975) and PA14-treated cells versus control (838). Each row of the plot is a gene and was colored according to the log2ratio of expression with red meaning up-regulation in relation to the controls and blue downregulation. The histogram shows the range of changes in a log2 scale. The data presented are for the three independent experiments combined. The heatmap was generated using the heatmap.2 function of the gplots package in R [47]. The dendrogram was generated using Euclidean distance and the "complete" agglomeration method.

**Figure 4:**
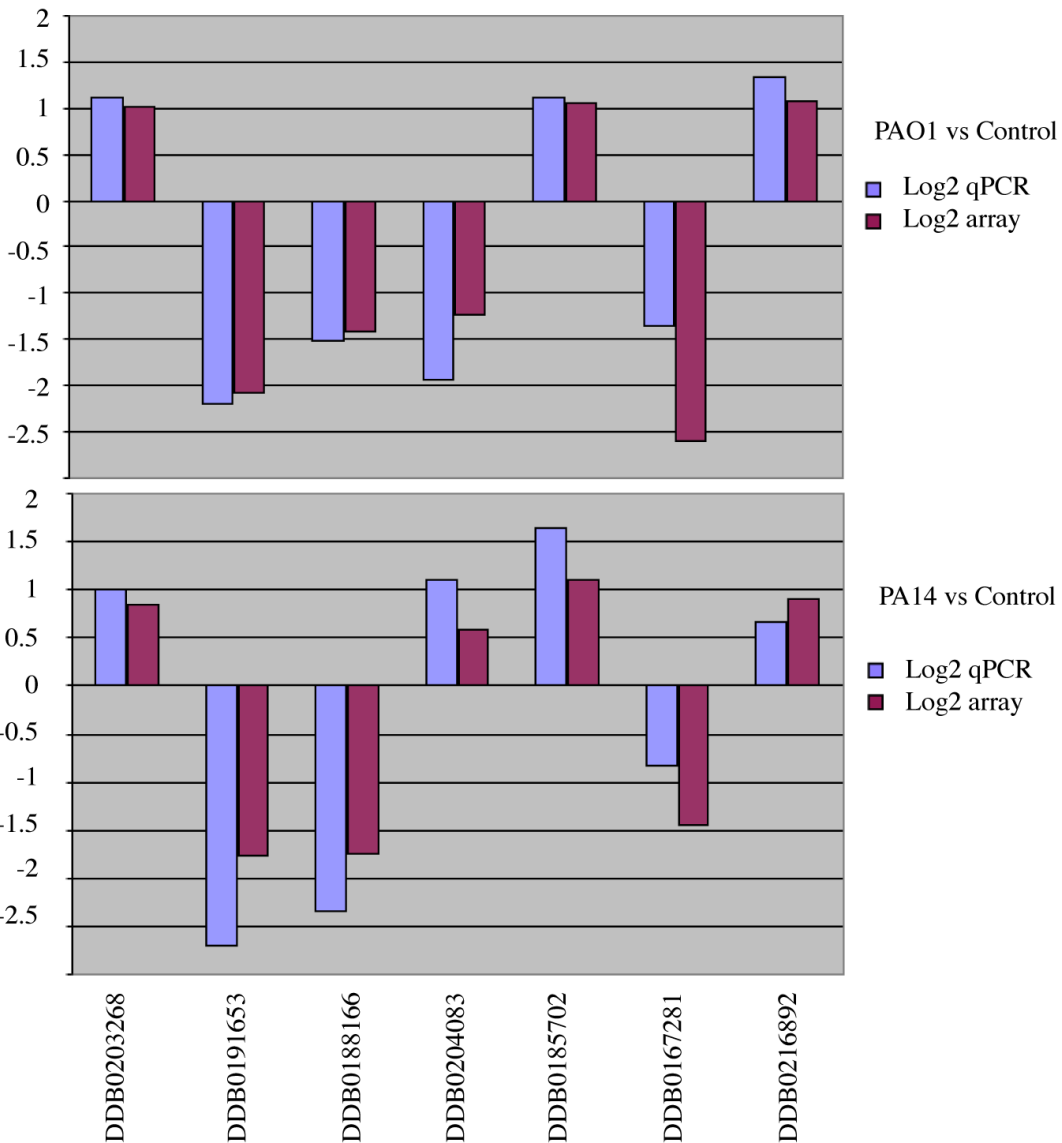
**Correlation of microaray and real-time PCR**. Real-time PCR measurements of the mRNA levels for seven representative genes whose expression were affected in the array. Upper panel shows a direct comparison of the changes in a log2 scale for PAO1 versus control and the lower panel shows the same genes for PA14 versus control. Blue bars corresponded to quantitative real time PCR and the purple bars to the array data. The array data and the real time PCR displayed are the combination of three independent biological experiments. The correlation coefficients were: R^2 ^= 0.87 for PAO1 and R^2 ^= 0.91 for PA14.

Little is known about the interplay between the host and the pathogen in terms of gene expression responses. We wanted to determine if there is a specific gene expression response of *D. discoideum *to their interaction with *P. aeruginosa*. *D. discoideum *cells were exposed to *P. aeruginosa *strains PAO1 and PA14 mixed with an excess of *K. aerogenes *in HL5 for 4 hours. *K. aerogenes *alone was used as a control to which the gene expression levels were compared. RNA was extracted from *D. discoideum *and used to study the global pattern of gene expression using whole-genome *D. discoideum *microarrays (see Additional file 1 for the complete data). Using a P < 0.05 cutoff, there were 752 genes whose expression was significantly different between the PAO1-treated cells and the controls and 624 genes between PA14-treated cells and controls (Table 1 summarizes the results at different P values and log-ratios). The heat map shown in Figure 3 indicates that the responses were broadly comparable between the two strains with very few genes oppositely altered in the two conditions. The differences in the gene expression are approximately in the range between four-fold repression and three-fold induction (log-ratios between of -2 to +1.5 as shown in the histogram of Figure 3). These results were validated by real time PCR of the same samples used for the transcriptomic assays, measuring the expression of 7 representative genes that were up-regulated or down-regulated in the different conditions. Figure 4 shows a good correlation between the data obtained from the microarray transcriptomic experiment as compared with that obtained by quantitative RT-PCR. Although the log-ratio changes in the gene expression showed some differences the overall trend were consistent, supporting the reliability of our data.

**Table 1:** Differential genes at p < 0.05 and different Log 2 ratios

	PAO1 vs Control	PA14 vs Control	PAO1+PA14 vs Control	PAO1 vs PA14
Log 2 ratio	**752**	**623**	**364 (Table 2)**	**169 (Table 3)**
(>+0.5 or <-0.5)	461 down 291 up	396 down 227 up	258 down 106 up	60 down 109 up
Log 2 ratio	**150**	**125**	**70**	**35**
(>+1 or <-1)	126 down 24 up	105 down 20 up	66 down 4 up	14 down 21 up

### Common and specific responses of *D. discoideum *to the infection with PAO1 and PA14 strains

**Figure 5:**
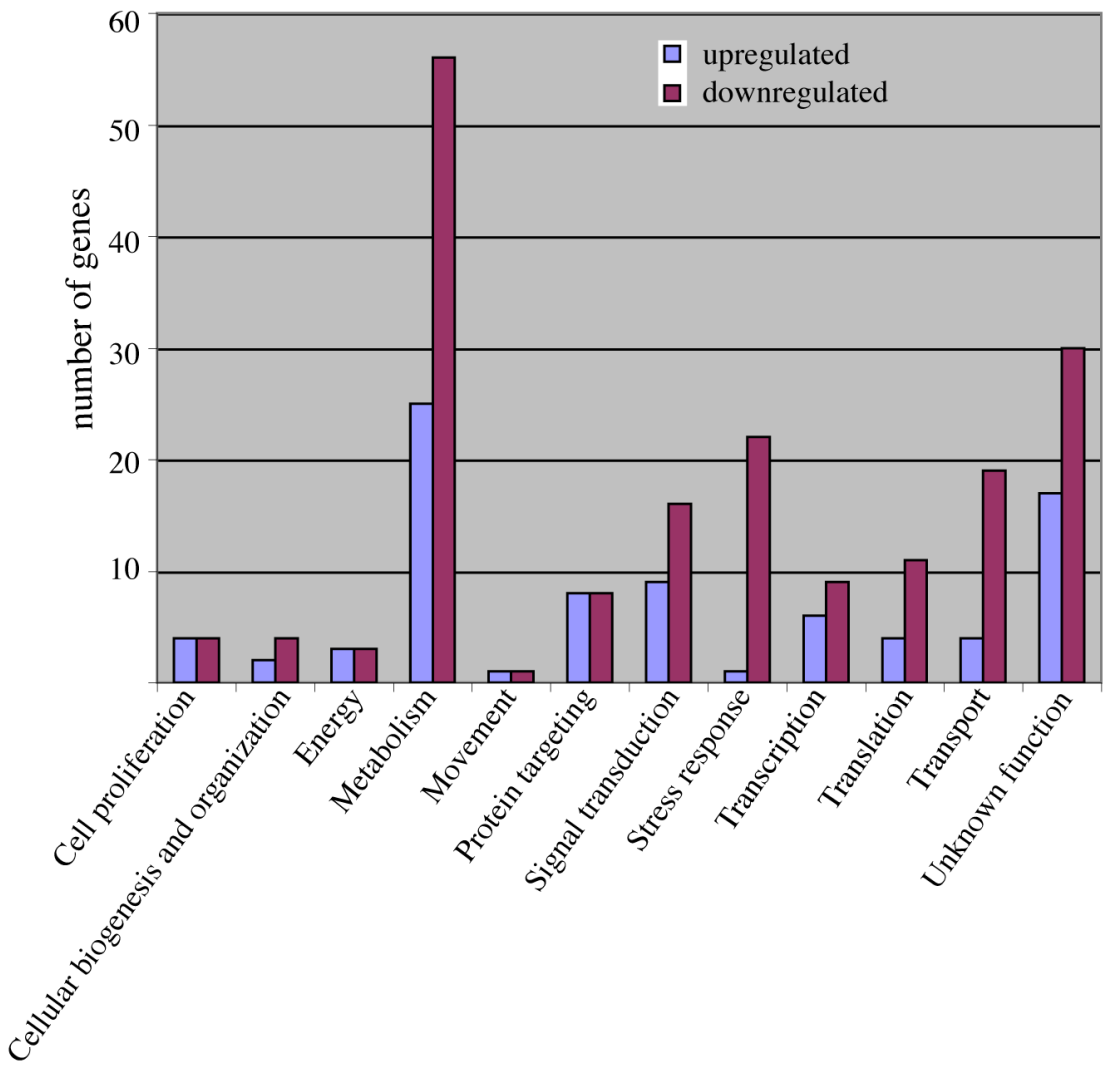
**Functional categories of the genes affected similarly by the exposure with PAO1 and PA14**. The genes whose expression was altered by PAO1 and PA14 were manually annotated (see Table 2) and grouped in functional categories. The size of the blue bars indicates the number of genes upregulated in each category related to the control and purple bars the number of genes downregulated.

As shown in Table 1 there were 364 genes that showed similar differential regulation with both bacterial strains compared with the controls (labeled as PAO1+PA14 vs control). We have considered in the analysis those genes showing differences in log-ratios that are higher than +0.5 or lower than -0.5. Interestingly the expression of another group of 169 genes (labeled as PAO1 vs PA14) was different depending on whether the infecting strain was PAO1 or PA14. We have studied in detail both groups by manual annotation and categorizing using the extended categorization for *D. discoideum *previously described [26]. Genes of unknown function and those showing weak homologies were not included in the list. Table 2 contains the genes that were similarly regulated upon infection with any of both strains, and in Figure 5 the genes are categorized by function (see Additional file 2 for the complete data). The first interesting conclusion from this experiment is the existence of a common transcriptional response that affects many different genes that are involved in a wide range of functions. The proportion of the genes that were downregulated by the treatment with both strains of *P. aeruginosa *is higher (258 genes) compared to those upregulated (106 genes). This difference is more evident in categories such as stress response and transport (Figure 5).

**Table 2:** Genes differentially expressed upon infection with PAO1 and PA14 versus *Klebsiella*

Gene ID	Gene function and name	Log 2 ratio: PAO1-C	PA14-C
**Cell proliferation**			
DDB0216882	Cyclin-dependent kinase regulatory	-0,89	-0,97
DDB0188449	cdc40, conserved splicing factor	0,68	0,78
DDB0205486	CDK family protein kinase	0,81	0,89
DDB0168249	cdk1, "cyclin-dependent_kinase, p34-cdc2_protein"	-0,78	-0,83
DDB0216532	cdk10;"putative_CDK_family_protein_kinase	0,91	1,08
DDB0185341	PP-loop family	-0,60	-0,70
DDB0205486	putative protein serine/threonine kinase, CDK family protein kinase	0,68	0,64
DDB0218360	PhoPQ-activated pathogenicity-related protein	-0,59	-0,57
**Cellular biogenesis and organization**			
DDB0189693	copA, coatomer protein complex alpha subunit	0,52	0,68
DDB0216892	lvsB;"BEACH_domain-containing_protein"	1,05	0,91
DDB0202609	Transport protein particle (TRAPP)	-0,77	-0,61
DDB0187558	putative mitochondrial import inner membrane translocase	-0,65	-0,65
DDB0186481	atg9, APG9, "autophagy_protein_9"	-0,88	-0,77
DDB0217942	Putative Mpv17/PMP22 family	-0,66	-0,93
**Energy**			
DDB0217090	Isocitrate lyase family	-0,80	-0,84
DDB0192001	ppkA, "poly_P_kinase, polyphosphate_kinase"	-0,65	-0,51
DDB0190821	sdhB, complex II, iron-sulfur protein (IP) subunit	0,74	0,53
DDB0190821	sdhB;"complex_II,(ubiquinone), succinic_dehydrogenase"	0,59	0,61
DDB0167662	similar to Coenzyme Q9	-0,71	-0,72
DDB0204006	AMPK beta-2 chain	0,52	0,73
**Metabolism**			
DDB0187528	cysteine dioxygenase	0,69	0,66
DDB0185702	hgd;"homogentisate_1,2-dioxygenase"	1,05	1,10
DDB0184361	Thiamine pyrophosphate enzyme	-0,57	-0,88
DDB0186120	gshA, "gamma_glutamylcysteine_synthetase, glutamate-cysteine_ligase"	-1,72	-0,94
DDB0167249	Aldehyde dehydrogenase	-0,77	-0,94
DDB0192169	alrA;"aldehyde_reductase, aldo-keto_reductase"	-0,54	-0,88
DDB0218652	alrB;"aldo-keto_reductase"	-0,60	-0,69
DDB0189745	alrC;"aldo-keto_reductase"	-0,84	-0,72
DDB0186332	alrE;"aldo-keto_reductase"	-1,23	-0,99
DDB0204015	D-Lactate dehydrogenase	0,92	0,65
DDB0187572	Endoglucanase_E_like	0,64	0,78
DDB0203268	glgB;"1,4-alpha-glucan_branching_enzyme, branching_enzyme"	1,02	0,85
DDB0187562	glk;glucokinase	0,69	0,56
DDB0217973	Gluconolactonase	0,85	0,82
DDB0202855	Glycoside hydrolase	-1,63	-1,45
DDB0202233	Glycosyl hydrolase family 7	-1,26	-1,38
DDB0167594	Glycosyl hydrolases family	0,60	0,56
DDB0204016	gpt10;"putative_glycophosphotransferase"	-0,69	-1,11
DDB0204037	Legume lectins beta-chain signature	-0,86	-1,16
DDB0206405	Mannosyl oligosaccharide glucosidase	-0,80	-0,68
DDB0205896	NAD-dependent epimerase/dehydratase family protein	-2,12	-1,73
DDB0204752	Phosphoglycerate mutase family	0,64	0,61
DDB0190464	Predicted kinase related to galactokinase and mevalonate kinase	0,52	1,02
DDB0186919	zinc-containing alcohol dehydrogenase	-2,09	-1,99
DDB0168737	zinc-containing alcohol dehydrogenase (ADH)	-1,35	-1,03
DDB0169356	carboxylic ester hydrolase	-1,03	-0,72
DDB0190523	CAS1;"cycloartenol_synthase"	0,85	0,88
DDB0185601	cutA;"fatty_acid_elongase_3-ketoacyl-CoA_synthase, long_chain_fatty_acid_elongase"	-0,93	-0,62
DDB0188166	delta-24-sterol methyltransferase	-1,42	-1,74
DDB0186908	eapA;"alkyl-dihydroxyacetonephosphate_synthase"	0,72	0,81
DDB0187604	enoyl-CoA hydratase/isomerase domain-containing protei	-0,65	-0,63
DDB0205302	Enoyl-CoA hydratase/isomerase family	-0,64	-0,68
DDB0205157	fadB, des5-2, "delta_5_fatty_acid_desaturase"	0,89	1,11
DDB0190288	fcsB, fatty acyl-CoA synthetase, long-chain-fatty-acid-CoA ligase	-1,45	-1,57
DDB0191679	GNS1/SUR4 family	-1,03	-0,59
DDB0205505	mfeB;"hypothetical_peroxisomal_multifunctional_enzyme_2"	-1,06	-0,65
DDB0191653	patatin family protein	-2,07	-1,77
DDB0218187	Perilipin family	-1,35	-1,04
DDB0184443	Saposin (B) Domains	0,59	0,59
DDB0190553	Similar to sterol-C4-methyl oxidase-like	-0,71	-0,91
DDB0217332	stearoyl-CoA desaturase	0,90	1,02
DDB0206478	allC;"allantoate_amidinohydrolase, allantoicase"	-1,12	-1,25
DDB0205700	cysteine desulfurase	-1,31	-1,26
DDB0169540	MOSC domain	-1,00	-0,67
DDB0187599	5 prime nucleotidase family	-0,90	-0,70
DDB0187063	3-methyl-2-oxobutanoate hydroxymethyltransferase	-1,83	-1,41
DDB0190860	adenine phosphoribosyltransferase	-0,98	-0,75
DDB0203073	adenosine deaminase-related growth factor	-1,35	-0,74
DDB0215237	ATP:D-ribose_5-phosphotransferase, ribokinase	-0,56	-0,51
DDB0206047	CTP synthase	-1,01	-1,20
DDB0187738	Cytidine and deoxycytidylate deaminase zinc-binding region	-0,76	-0,73
DDB0191911	putative RNA methylase	-1,01	-1,21
DDB0185785	Putative RNA methylase family	-0,59	-0,63
DDB0219236	pyrK, cytidylate kinase	-0,98	-0,71
DDB0217423	rnrB_2, ribonucleotide reductase small subunit	-0,79	-0,84
DDB0215284	tRNA/rRNA methyltransferase SpoU family protein	-0,77	-0,93
DDB0186269	Thioredoxin family	-1,41	-1,91
DDB0206431	FAD binding domain	-1,63	-1,13
DDB0187958	gchA, "GTP_cyclohydrolase_I"	-0,91	-0,89
DDB0185963	Oxysterol-binding protein	-0,55	-0,75
DDB0205608	pks18, putative polyketide synthase	0,54	0,60
DDB0168380	pks5, putative polyketide synthase	0,57	0,99
DDB0219613	stlB, putative polyketide synthase	0,68	1,05
DDB0186173	Histidine acid phosphatase	-0,79	-0,80
DDB0184156	Acetyltransferase (GNAT) family	0,81	0,78
DDB0205937	Aldehyde dehydrogenase family	1,02	0,81
DDB0183800	dihydrolipoamide_dehydrogenase	-0,60	-0,57
DDB0188526	FAD binding domain	-1,14	-0,93
DDB0169374	haloacid dehalogenase-like hydrolase	-1,26	-1,45
DDB0204714	hemA, ALAS, "5-aminolevulinate_synthase, ALA_synthase"	1,00	0,99
DDB0187575	monooxygenase, FAD-binding	-1,30	-1,14
DDB0203608	NADH:flavin oxidoreductase/NADH oxidase domain-containing protein	-2,99	-2,49
DDB0186877	Predicted hydrolases or acyltransferases	-0,70	-0,83
DDB0203708	Putative dehydrogenase domain	0,60	0,66
DDB0186921	Putative quinone oxidoreductase	-0,68	-0,80
DDB0218378	selD, selenophosphate synthase	0,83	0,85
DDB0219578	short chain dehydrogenase	-0,56	-0,76
DDB0168766	short chain dehydrogenase	0,75	0,90
DDB0201995	Short-chain alcohol dehydrogenase	-0,64	-0,69
DDB0191047	Sucrolytic enzyme/ferredoxin homolog protei	-0,76	-0,55
DDB0205223	Ubiquinone biosynthesis protein	1,02	0,99
**Movement**			
DDB0190345	Actin	-0,62	-0,64
DDB0216677	tubB;"beta_tubulin"	0,78	0,66
**Protein targeting**			
DDB0189280	CLN3 protein;Major Facilitator Superfamily	-1,38	-0,81
DDB0186130	pigF, phosphatidylinositol glycan, class Fphosphoethanolamine N- methyltransferase family	-1,13	-1,33
DDB0187271	Protein prenyltransferase, alpha subunit	-0,69	-0,66
DDB0187195	Ubiquitin family protein	-0,68	-0,56
DDB0217546	vps13B, vacuolar protein sorting-associated protein	0,80	0,57
DDB0205767	Importin-beta N-terminal domain	-1,39	-0,91
DDB0219696	CSN3, COP9 signalosome complex subunit 3	0,86	0,56
DDB0188097	mppB;"mitochondrial_processing_peptidase_beta_subunit"	0,85	0,93
DDB0188792	npl4, nuclear protein localization 4	-1,46	-1,26
DDB0189322	Peptidase family M41	0,66	0,82
DDB0204548	putative E3 ubiquitin ligase	0,56	0,88
DDB0203213	Putative serine protease	0,57	0,64
DDB0190305	RING-finger-containing ubiquitin ligase	-1,71	-2,23
DDB0191910	sigB, GP63, orfGP63", "leishmanolysin_family_protein, peptidase	-1,44	-1,19
DDB0219558	usp12, putative ubiquitin carboxyl-terminal hydrolase (UCH)	0,81	0,50
DDB0188490	usp40, putative ubiquitin carboxyl-terminal hydrolase (UCH)	0,83	0,60
**Signal transduction**			
DDB0167328	ArfGAP, Arf GTPase activating protein	0,58	0,67
DDB0187828	gacX, RacGAP	0,62	0,52
DDB0217797	gpaG, "G-protein_subunit_alpha_7"	-0,93	-0,77
DDB0205484	GTPase-activator protein for Ras-like	0,58	0,69
DDB0202545	rabX;"Rab_GTPase"	-0,59	-0,76
DDB0186244	abkD, AdckB2, "putative_ABC1_family_protein_kinase"	0,80	0,88
DDB0217600	nek3, putative protein serine/threonine kinase	0,55	0,56
DDB0203684	tyrosine kinase-like	0,53	0,56
DDB0189806	vwkA;"protein_serine/threonine_kinase"	-0,77	-0,67
DDB0205355	Calcineurin-like phosphoesterase	-1,27	-0,86
DDB0218779	pdsA, "PDE, pde1, pdeA", "cAMP_phosphodiesterase"	0,79	1,40
DDB0204820	rabS;"Rab_GTPase"	-0,80	-0,87
DDB0190872	NLI interacting factor-like phosphatase	-0,55	-0,60
DDB0185382	Protein phosphatase 5, catalytic subunit	-0,68	-0,62
DDB0186390	protein tyrosine phosphatase	-0,85	-0,74
DDB0218065	ptpB, DdPTPa, "phosphotyrosine_phosphatase_ptp2	-1,20	-1,01
DDB0189698	Tyrosine specific protein phosphatases family	-0,76	-0,89
DDB0203756	G-protein-coupled receptor (GPCR) family protein	0,69	0,99
DDB0189216	gacD, RacGAP	-0,63	-0,58
DDB0217433	Regulator of G protein signaling	0,90	0,72
DDB0169375	cGMP-specific phosphodiesterase	-0,83	-0,79
DDB0167494	plbF, PLB, "phospholipase_B-like"	-1,17	-1,01
DDB0168860	sgkA, "SK, SPHK", "sphingosine_kinase"	-0,97	-0,79
DDB0167227	Cytochrome b5-like Heme/Steroid binding domain	-0,71	-1,57
DDB0216720	Tetraspanin family	-0,73	-0,68
**Stress response**			
DDB0202483	AhpC/TSA family	-0,51	-0,68
DDB0203727	AhpC/TSA family protein. Thioredoxin-like	-1,25	-1,89
DDB0203727	AhpC/TSA family protein. Thioredoxin-like	-1,11	-2,00
DDB0205904	AhpC/TSA family protein. Thioredoxin-like	-1,10	-1,63
DDB0168230	Cytochrome P450	0,61	0,67
DDB0217979	cytochrome P450 family protein	-1,11	-1,24
DDB0187276	cytochrome P450 family protein	-0,73	-0,88
DDB0186118	cytochrome P450 family protein	-0,54	-0,51
DDB0167587	Glutathione S-transferase	-2,24	-2,02
DDB0218804	Glutathione S-transferase	-1,07	-0,86
DDB0185602	putative FMN-dependent NAD(P)H:quinone reductase	-0,66	-0,93
DDB0168563	putative glutathione S-transferase	-0,64	-1,23
DDB0201962	Ku70-binding family protein	-0,60	-0,51
DDB0191833	TFIIH4, "TFIIH_subunit, general_transcription_factor_IIH,_polypeptide_4"	-0,81	-0,90
DDB0204089	NUDIX hydrolase family	-0,89	-1,14
DDB0185428	Strictosidine synthase	-1,74	-1,01
DDB0169033	trap1, Dd-trap1, "TNF_receptor-associated_protein", member of the HSP90 fam	-1,93	-1,56
DDB0188234	Chaperone clpB	-1,00	-1,25
DDB0192088	heat shock cognate protein	-0,82	-0,99
DDB0192086	heat shock protein, 70 kDa heat shock protein	-0,94	-1,18
DDB0169051	Hsp20/alpha crystallin family	-1,13	-1,30
DDB0169044	Hsp20/alpha crystallin family	-1,07	-1,12
DDB0169207	hspG12, heat shock protein Hsp20 domain-containing protein	-0,72	-0,86
**Transcription**			
DDB0204405	CRTF;"transcription_factor"	0,97	1,15
DDB0167879	IWS1 C-terminus	0,70	0,69
DDB0205969	snd1, tudor domain-containing protein	0,66	0,79
DDB0188840	TFIIAL, "transcription_factor_IIA"	-0,79	-0,55
DDB0167865	ddx52, DEAD/DEAH box helicase	-0,55	-0,55
DDB0184074	ddx6, DEAD/DEAH box helicase	0,62	0,66
DDB0184228	DEAD/DEAH box helicase	-0,63	-0,60
DDB0206136	myb domain-containing protein	0,59	0,58
DDB0189583	rpmA;"DNA-dependent_RNA_polymerase"	-1,33	-1,17
DDB0186101	pwp2, ortholog of H. sapiens and S. cerevisiae PWP2	-0,80	-0,88
DDB0192008	rpa2, RNA polymerase I, second largest subunit	-0,98	-1,13
DDB0218008	rpc4;"putative_RNA_polymerase_III_subunit"	-0,58	-0,55
DDB0216877	tRNA pseudouridine synthase	-0,56	-0,65
DDB0204724	DNA helicase TIP49, TBP-interacting protei	0,63	0,64
DDB0219410	pirin-like protein	-0,99	-0,81
**Translation**			
DDB0167043	Ribosomal protein L10	-0,86	-1,37
DDB0190639	Fibrillarin	-0,65	-0,84
DDB0184302	Mitochondrial small ribosomal subunit Rsm22	-0,70	-0,72
DDB0205674	MPP10, U3 small nucleolar ribonucleoprotein	-0,77	-0,66
DDB0201601	mrpl11, S60 ribosomal protein L11, mitochondrial	-0,91	-1,26
DDB0204554	Ribosomal protein L28	-0,50	-0,69
DDB0188692	Ribosomal protein S8e	-0,94	-0,79
DDB0183814	Ribosomal RNA processing protein 4	0,58	0,56
DDB0188661	rps9, "rp1024, v12", "40S_ribosomal_protein_S9	0,60	0,70
DDB0219852	u3 small nucleolar RNA interacting protein 2, putative	-0,75	-0,63
DDB0191852	eukaryotic translation initiation factor 3 subunit 5	0,59	0,54
DDB0203843	Eukaryotic translation initiation factor 6 (EIF-6)-like protein	-0,96	-0,64
DDB0189529	gfm2, mitochondrial translation elongation factor G	-0,59	-0,50
DDB0202851	NMD3 family	-0,95	-0,97
DDB0168814	aspartyl-tRNA_synthetase	0,70	0,98
**Transport**			
DDB0217304	ABC transporter AbcG17	-1,58	-1,44
DDB0167281	ABC transporter mdrA2	-2,65	-1,44
DDB0167281	ABC transporter mdrA2	-2,26	-1,45
DDB0191940	abcB2;"ABC_transporter_B_family_protein"	0,87	0,93
DDB0188931	abcE1;"RNaseL_inhibitor-like_protein, non-transporter_ABC_protein"	0,61	0,74
DDB0189332	amino acid permease family protein	-0,72	-0,87
DDB0168564	Amino acid/polyamine transporter	-1,28	-1,06
DDB0190286	mcfF, Mitochondrial carrier protein	-0,99	-0,86
DDB0216936	mftA;"carrier_protein_RIM"	-0,91	-1,05
DDB0188529	nucleoporin family protein	-0,94	-0,72
DDB0189222	ccsA, copper chaperone for superoxide dismutase	-0,69	-0,62
DDB0205129	Co/Zn/Cd efflux system component	-0,56	-0,71
DDB0202441	nhe1, DdNHE1, "Na-H_exchanger, sodium/hydrogen_exchanger"	-0,77	-0,59
DDB0168533	porA;porin	0,68	0,59
DDB0218156	P-type cation-transporting ATPase	-0,69	-0,57
DDB0189480	mcfT, mitochondrial substrate carrier family protein	-0,74	-0,86
DDB0202337	Nodulin, Major Facilitator Superfamily	-0,60	-0,68
DDB0185520	Nucleoside transporter	-0,53	-0,56
DDB0203447	Sugar (and other) transporter	1,01	0,71
DDB0168979	Sugar transport proteins signature 1	-0,78	-1,28
DDB0189650	sodium/potassium-transporting ATPase alpha chain 2	-1,48	-0,81
DDB0190036	Major Facilitator Superfamily	-1,13	-1,05
DDB0205693	Major Facilitator Superfamily	-1,33	-1,08

**Figure 6:**
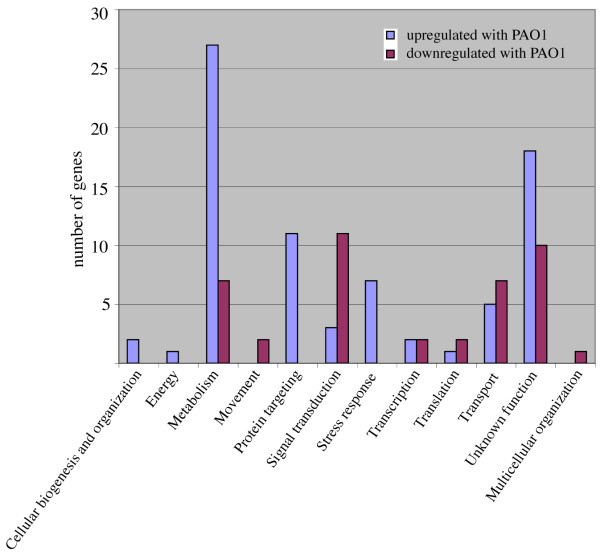
**Functional categories of the genes differentially affected by the exposure to PAO1 and PA14**. The genes whose expression was differentially altered in PAO1 versus PA14 were manually annotated (see Table 3) and grouped in functional categories. The size of the blue bars indicates the number of genes upregulated in PAO1 versus PA14 in each category and purple bars the number of genes downregulated in PAO1 versus PA14.

Table 3 displays the *D. discoideum *genes whose expression changed differentially between PAO1 and PA14 infection and Figure 6 shows the number of genes in each category (see Additional file 3 for complete data). In general a higher proportion of the genes showed a higher level of expression by PAO1 infection (109 genes) when compared with the levels observed by PA14. On the other hand, 60 genes behaved oppositely showing lower levels of expression upon exposure to PAO1 compared to those levels obtained after PA14 infection. Interestingly, all the genes represented in the categories stress-response and protein targeting had a higher level of expression in the cells exposed to PAO1 compared to PA14. The behavior of these genes in comparison with the control is also displayed in Table 3.

**Table 3:** Genes differentially expressed upon infection with PAO1 versus PA14

Gene ID	Gene function and name	Log2 ratio: PAO1-14	PAO1-C	PA14-C
**Cellular Biogenesis and organization**				
DDB0187116	vps13A, vacuolar protein sorting-associated protein	0,81	0,67	-0,13
DDB0189855	vps46, Vacuolar Protein Sorting	0,83	0,59	-0,24
**Energy**				
DDB0204335	cxgE, "cox7E, coxVIIe", "cytochrome_c_oxidase_subunit_VII_E"	1, 20	0,80	-0,40
**Metabolism**				
DDB0190752	diaminopimelate epimerase	1,08	0,02	-1,06
DDB0204319	Hydroxymethyltransferase	0,63	0,43	-0,20
DDB0168738	putative arginine deiminase	-0,62	-0,78	-0,16
DDB0205389	acly, ATP citrate lyase	0,89	0,75	-0,15
DDB0169357	methylenetetrahydrofolate dehydrogenase	0,78	0,21	-0,57
DDB0187393	NAD-dependent epimerase/dehydratase family protein	-0,91	-1,18	-0,28
DDB0205386	putative ATP citrate synthase	0,96	0,79	-0,17
DDB0205339	rpe;"ribulose_phosphate_3-epimerase"	0,78	0,43	-0,35
DDB0187942	Short-chain alcohol dehydrogenase of unknown specificit	0,70	0,43	-0,27
DDB0187544	smlA	1,43	1,00	-0,43
DDB0217455	zinc-containing alcohol dehydrogenase (ADH)	-1,21	-1,16	0,06
DDB0217374	zinc-containing alcohol dehydrogenase (ADH)	-1,02	-1,37	-0,35
DDB0190948	acid ceramidase-like protein	-0,87	-0,31	0,56
DDB0188248	Acyltransferase	0,64	0,16	-0,48
DDB0219652	cinB, "esterase/lipase/thioesterase_domain-containing_protein	0,92	1,28	0,36
DDB0189754	esterase/lipase/thioesterase domain-containing protein	1,07	1,07	0,00
DDB0185740	esterase/lipase/thioesterase domain-containing protein	1,08	1,27	0,20
DDB0184141	Phosphate acyltransferases	0,55	-0,26	-0,82
DDB0167446	pks16, putative fatty acid synthase	1,19	1,81	0,63
DDB0189182	Putative esterase/lipase/thioesterase	0,99	1,43	0,44
DDB0191907	dUTP diphosphatase	0,61	0,14	-0,47
DDB0217901	purH, AICAR transformylase/IMP cyclohydrolase	0,89	0,34	-0,55
DDB0167009	pyr4;"dihydroorotate_dehydrogenase, dihydroorotate_oxidase"	0,68	0,18	-0,50
DDB0217842	rpiA;"ribose-5-phosphate_isomerase"	0,63	0,42	-0,21
DDB0189571	Sulfite reductase, alpha subunit (flavoprotein)	-0,89	-0,09	0,79
DDB0202318	Cyclopropyl sterol isomerase	0,73	0,31	-0,42
DDB0167227	Cytochrome b5-like Heme/Steroid binding domain	0,86	-0,71	-1,57
DDB0168923	dihydropteridine reductase	0,60	0,14	-0,47
DDB0185998	ERG24, Ergosterol biosynthesis	0,59	-0,02	-0,61
DDB0219255	Fe(II) oxygenase superfamily	1,14	0,91	-0,23
DDB0217308	Predicted iron-dependent peroxidase	-0,55	-0,12	0,43
DDB0192180	putative O-methyltransferase	0,56	0,71	0,15
DDB0202301	putative SAM dependent methyltransferase	0,88	0,94	0,05
DDB0167345	short-chain dehydrogenase/reductase (SDR) family protein	0,73	0,56	-0,17
**Movement**				
DDB0188280	myoB, "DMIB, abmB", "myosin_IB"	-0,85	-0,30	0,55
DDB0167337	myoD, DMID, "myosin_ID_heavy_chain"	-0,81	-0,76	0,05
**Multicellular organization**				
DDB0216906	comC;"FIBROSURFIN_PRECURSOR"	-0,65	-0,39	0,26
**Protein destination**				
DDB0189735	homolog to co-chaperone p23	0,56	0,45	-0,11
DDB0187409	Acetyltransferase (GNAT) family	0,57	0,33	-0,24
DDB0189799	SET domain-containing protein	0,52	0,33	-0,19
DDB0202482	26S proteasome non-ATPase regulatory subunit 9	0,78	1,25	0,47
DDB0219654	Cysteine proteinase 1 precursor	0,70	0,61	-0,09
DDB0167298	Dipeptidyl aminopeptidase	0,51	-0,07	-0,59
DDB0190542	Probable proteasome subunit beta type 3	0,57	-0,06	-0,63
DDB0216902	prtA, M3L, "proteosomal_alpha-subunit_M3"	0,93	0,94	0,01
DDB0216901	prtB, M3R, "proteosomal_alpha-subunit_7-1"	1,37	1,30	-0,06
DDB0186869	small ubiquitin-like protein	0,61	0,33	-0,28
DDB0217344	ubiquitin-like domain containing CTD phosphatase	0,52	0,29	-0,23
**Signal transduction**				
DDB0169410	gpaB, "Ga2, Galpha2, gpa2", "G-protein_subunit_alpha_2"	-0,82	-0,55	0,27
DDB0190318	rab1C;"Rab_GTPase"	0,87	0,76	-0,10
DDB0202066	pakC, STE20 family protein kinase	-0,61	-0,18	0,43
DDB0169250	putative protein serine/threonine kinase	-0,87	-0,58	0,29
DDB0205782	roco6;"putative_protein_serine/threonine_kinase	-0,61	-0,48	0,14
DDB0167076	sepA, putative protein serine/threonine kinase	-0,57	-0,31	0,26
DDB0217465	fslH, G-protein-coupled receptor (GPCR) family protein	0,71	0,24	-0,46
DDB0205174	grlF, GABA-B receptor-like protein	-1,41	-1,54	-0,12
DDB0168770	grlJ, GABA-B receptor-like protein	-0,63	-0,96	-0,32
DDB0204083	grlL, GABA-B receptor-like protein	-1,84	-1,25	0,60
DDB0229801	grlQ, G-protein-coupled receptor (GPCR) family protein	0,77	0,98	0,21
DDB0167432	gacFF, RacGAP	-0,57	0,08	0,66
DDB0167384	putative guanine nucleotide exchange factor (GEF)	-0,70	-0,01	0,70
DDB0167541	dpoA;"prolyl_oligopeptidase"	-0,66	-0,11	0,55
**Stress response and cell rescue**				
DDB0218719	AhpC/TSA family protein	0,82	0,76	-0,05
DDB0205882	AhpC/TSA family protein. Thioredoxin-like	0,75	0,57	-0,18
DDB0217383	Cytochrome P450	1,16	0,42	-0,74
DDB0168563	putative glutathione S-transferase	0,59	-0,64	-1,23
DDB0167491	alyA;lysozyme	0,63	0,73	0,10
DDB0167489	alyB;lysozyme	0,69	0,75	0,07
DDB0167490	alyC;lysozyme	0,71	0,87	0,16
**Transcription**				
DDB0206051	member of NOD protein family	1,05	0,90	-0,15
DDB0202276	srfC;"putative_MADS-box_transcription_factor"	-0,90	-0,30	0,60
DDB0217613	wrky1;"putative_WRKY_transcription_factor"	1,04	1,11	0,07
DDB0167422	putative histone acetyltransferase	-0,64	-0,55	0,09
**Translation**				
DDB0201621	mrps2, ribosomal protein S2, mitochondrial	0,75	0,49	-0,27
DDB0218535	Eukaryotic elongation factor 1 (EF1) alpha subfamily	-0,83	-0,18	0,65
DDB0167339	tRNA-ribosyltransferase	-0,52	-0,56	-0,04
**Transport facilitation**				
DDB0187089	abcC5;"ABC_transporter_C_family_protein"	-1,12	-0,68	0,44
DDB0218568	Copper-transporting P-type ATPase	-1,14	-0,60	0,54
DDB0204460	patA, PAT1, "Ca2+-ATPase, P-type_ATPase"	-0,93	-0,15	0,78
DDB0205031	potassium channel tetramerization domain-containing protein	0,92	0,84	-0,08
DDB0217251	P-type cation-transporting ATPase	-2,09	-1,46	0,63
DDB0186223	P-type cation-transporting ATPase	-0,69	-0,40	0,29
DDB0167361	vatE;"vacuolar_H+-ATPase_E_subunit"	0,54	0,47	-0,08
DDB0217699	phospholipid-translocating P-type ATPase family protein	-0,67	-0,63	0,04
DDB0187945	Sugar (and other) transporter	-0,90	-0,78	0,11
DDB0206579	mcfQ, mitochondrial substrate carrier family protein	0,65	0,78	0,13
DDB0192172	mcfZ, mitochondrial substrate carrier family protein	0,73	0,92	0,18
DDB0183815	Mitochondrial carrier protein	0,55	0,81	0,26

## Discussion

*P. aeruginosa *is able to infect *D. discoideum *cells using several virulence traits that are similar to those used to infect mammalian cells and other hosts [18]. The clinical *P. aeruginosa *isolates PA14 and PAO1 have been used independently to study the infection of *Dictyostelium *by *Pseudomonas *in two different laboratories [20,21]. However, no direct comparison had been reported so far between these strains in this pathogenicity model. We now report that PA14 is indeed more virulent in *D. discoideum *using different plating assays. Since *P. aeruginosa *is phagocytosed at much lower rate than the non-pathogenic *K. aerogenes *(commonly used to grow *Dictyostelium*) [20], the assays were designed to provide sufficient food to *D. discoideum *to avoid cell starvation. Thus, *K. aerogenes *was always used in excess together with the pathogenic strains. In the first assay (Figure 1) a nutrient plate was used to allow the growth of bacteria and *D. discoideum *simultaneously. Under these conditions the presence of PA14 inhibits *D. discoideum *growth to a greater extent than PAO1. To avoid differences in the growth rates between bacteria that might alter their final proportions, a non-nutrient assay was performed (Figure 2). In these experiments, *D. discoideum *feed on bacteria that have been previously grown and deposited at different proportions in non-nutrient agar. Interestingly, PAO1 is not virulent in this condition suggesting that bacterial growth is necessary for the expression of the virulence in this strain. However, even in these conditions PA14 is capable of inhibiting *D. discoideum *growth. Some studies have suggested that PA14 pathogenicity is multifactorial and required the action of multiple virulence mechanisms [15,27]. These differences between strains prompted us to study the transcriptional profile of *D. discoideum *upon infection with PAO1 and PA14 to gain insights not only into the possible common transcriptional response but also into any specific response that could explain the observed differences in their virulence. Pilot experiments showed that 4 hours of exposure of *D. discoideum *cells to either *Pseudomonas aeruginosa *strains did not result in any apparent cell death or change in cell morphology (data not shown). Since we wanted to study the early transcriptional response we chose this short time of exposure to avoid changes due to cell death. The existence of a rapid gene expression response between 1–6 hours upon exposure of *D. discoideum *cells to *Legionella*, an intracellular pathogen, has also been described [28].

Our results show the existence of a common transcriptional response to the infection with *P. aeruginoa *PAO1 or PA14 that affects 364 genes grouped in many different cellular functions. The complexity of the observed transcriptional changes could be the result of the induction of *D. discoideum *defensive responses or triggered by *P. aeruginosa *to make a less hostile cell environment that would support a better survival of the pathogen. In this scenario downregulation of genes involved in stress response might be beneficial for a successful infection. Interestingly, we have observed a clear down-regulation of genes dedicated to stress in the common response to PAO1 and PA14 but also in the specific response to the more virulent strain PA14. For example the gene coding for Strictosidine synthase (DDB0185428) which is involved in the synthesis of alkaloids related to defense mechanisms in plants [29], Trap1 (DDB0169033) that plays a central role in cell cycle regulation and differentiation [30] or the genes coding for lysozymes involved in bacterial degradation (DDB0167491) [31], to mention just a few.

Besides stress response other categories are affected by an overall downregulation such as metabolism, translation and transport facilitation. A subcategorization of the genes included in metabolism (see supplementary table 2) showed that all the genes coding for proteins involved in nucleotide metabolism were downregulated in the common response suggesting an effect on cell proliferation. Moreover, a number of other downregulated genes are directly involved in cell growth as demonstrated functionally in previous studies. This is the case for example of DDB0192001 (ppkA, polyphosphate kinase), whose disruption leads to reduced growth on bacteria [32,33], DDB0186120 (gcsA, glutamylcysteine synthetase), which is essential for cell growth as mutants in the gene are not viable in the absence of glutathione [34]. DDB0168860 (sgkA, sphingosine kinase) that is involved in cell proliferation [35], among others that have been annotated in Supplementary Table 2, 3.

Two different expression microarray analysis in mammals upon infection with *Pseudomonas aeruginosa *have been reported. In the first report epithelial cells were exposed to the pathogen for 3 hours, a short exposure similar to our experimental design. Unfortunately the number of genes represented in the array was very limited (1500 cDNAs) [36]. Only 22 genes were differentially regulated and we have not found any homologous gene in common. The other work reported the analysis of *Pseudomonas aeruginosa *corneal infection using an oligonucleotide microarray [37]. This experiment is not directly comparable to ours since a long exposure to the pathogen (1 day) was performed to assure an infection process. As a consequence most of the regulated genes were associated with the immune response and apoptosis, aspects that are not present in *Dictyostelium*.

*D. discoideum *is also susceptible to the infection by *Legionella pneumophila*, a facultative intracellular parasite, which uses different infective mechanisms from *P. aeruginosa*. It is important to note here that the transcriptional response of *D. discoideum *upon infection with *Legionella *[28] was essentially different to the one we report for *P. aeruginosa*. Only 8 genes were found to be altered in both experiments (DDB0186332, DDB0219578, DDB0167879, DDB0205386, DDB0185740, DDB0167345, DDB0201617, DDB0202615). This indicates that the host response is rather specific of the type of infection and the bacterial pathogen involved. Nevertheless, some responses can be also common. For instance, DDB0202615 (nramp1, natural resistance-associated macrophage protein) whose expression is downregulated in PAO1 and PA14, plays an important role in Legionella infection since the null mutant has increased sensitivity to the infection [38]. Nramp1 transports metal cations out of the phagolysosome in an ATP-dependent process. This activity is believed to be necessary to avoid the growth of intracellular pathogens and might also contribute to the efficient killing of other bacterial pathogens.

The variety of genes whose expression is altered by *P. aeruginosa *infection suggests a complex scenario in which a combined downregulation of the expression of some of the mentioned genes might affect *D. discoideum *fitness thus favoring the infection. The precise role of these genes in the pathogenesis and the mechanisms that regulates their expression will promote further investigation.

## Conclusion

Our results showed that *P. aeruginosa *PA14 is more virulent than PAO1 in the *D. discoideum *model using different plating assays. The transcriptional responses of *D. discoideum *infected by either *P. aeruginosa *PAO1 or PA14 were analyzed by whole-genome microarrays and the expression of 364 genes changed similarly upon infection with any of both strains as compared with the control. Effects on metabolism, signaling, stress response and cell cycle can be inferred from the genes affected. Interestingly there were 169 genes differentially regulated between PAO1 and PA14, and this differential response might contribute to the different virulence behavior displayed by these two model strains. This is a starting point to begin to understand the complex relationships between environmental opportunistic pathogens and their natural hosts. Besides, our data support the idea that the host responses to different isolates of the same bacterial pathogen are largely different, thus indicating that the crosstalk between the pathogen and its host is more specific and more complex than previously thought.

## Methods

### *D. discoideum *growth and plating assays

Dictyostelium AX4 cells were grown axenically in HL5 medium (14 g/l tryptone, 7 g/l yeast extract, 0.35 g/l Na_2_HPO_4_, 1.2 g/l KH_2_PO_4_, 14 g/l glucose, pH 6.5) or in association with *Klebsiella aerogenes *on SM plates (10 g/l glucose, 1 g/l yeast extract, 10 g/l peptone, 1 g/l MgSO_4_·6H_2_0, 1.9 g/l KH_2_PO_4_, 0.6 g/l K_2_HPO_4_, 20 g/l agar, pH 6.5) [39].

For the nutrient SM-plating assay *Pseudomonas aeruginosa *(PAO1 and PA14) and *Klebsiella aerogenes (KA) *were grown overnight in LB. After washing, bacteria were resuspended with PDF (20 mM KCl; 9 mM K_2_HPO_4_, 13 mM KH_2_PO_4_, 1 mM CaCl_2_; 1 mM MgSO_4_; pH: 6.4) and the optical density (OD) determined at 600 nm. After adjusting to 0.5 OD units, 300 μl of *Klebsiella *and *Pseudomonas *at the indicated proportions were plated in SM-agar plates with approximately 100 *D. discoideum *cells.

For non-nutrient plating assay bacteria were grown as before, washed and resuspended in PDF (at an OD of 15 units at 600 nm. 100 μl of bacteria at the indicated proportions were mixed with *D. discoideum *cells and deposited in a drop over a PDF-based non-nutrient agar plates.

### Microarrays

*Dictyostelium *cells (5 × 10^7 ^cells) were deposited in 10 ml of HL5 (without antibiotics) in shaking culture and exposed during 4 hours to 1.0 OD (approximate multiplicity of infection: 1000) of *Klebsiella aerogenes *as a control or to a mixture of *Pseudomonas aeruginosa *(PAO1 and PA14) and *Klebsiella *(used in excess to provide similar conditions of food supply). The proportion of *Klebsiella *to *Pseudomonas *(either PAO1 or PA14) was 7:3. Previous experiments showed that these proportions are adequate for a clear inhibition of *Dictyostelium *growth in plating assays (see the results section). *Dictyostelium *cells were separated from the bacteria by gentle centrifugation (twice at 1000 rpm, 5 minutes) and RNA isolated by Trizol (Life Technologies) according to manufacturer's instructions. Three independent biological experiments were performed making a total of three independent treatments for each condition (*Klebsiella *control, PAO1, and PA14). RNA from the three treatments were paired in all three combinations (Control/PAO1; Control/PA14 and PAO1/PA14) and hybridized to three different arrays. The same was performed for the other two biological replicates making a total of 9 microarrays hybridized. One of the three biological replicates was hybridized in the opposite dye orientation to the other two. The arrays, and protocols for labelling, hybridisation and scanning were as previously described [40]. Background fluorescence was subtracted [41], linear models were fitted and the significance of apparent changes in expression was assessed using limma [42,43]. The data were normalised within each array with the printtip loess algorithm to counteract scanning and spatial biases, and further between each array to normalise mean absolute deviations using the 'scale' algorithm [44].

Preliminary analysis using ANOVA methods indicated that many genes had significant differences in expression between treatments, so we proceeded to examine each pairwise contrast in turn. We filtered out less reliable data by selecting genes with p-values adjusted for multiple testing [45] less than 0.05, making use of the moderated t statistics calculated by the eBayes function of limma. Since many genes passing this cutoff showed small changes in expression, we filtered further by absolute log2ratio. The commonly-used cutoff of greater than 2-fold change would have excluded a large number of genes that appeared to change in expression quite consistently, so we used the less stringent criterion of absolute log2ratio >0.5. A lower log2ratio would have included genes with differences in expression too small to be corroborated by other methods. The array design is available from ArrayExpress [46] under the accession A-SGRP-3. The array experiment was deposited in the ArrayExpress database under the accession E-TABM-464.

### Quantitative PCR

The same RNA samples subjected to microarray study were used as templates for retrotranscription with High Capacity cDNA Reverse transcription kit (Applied Biosystems) using 250 ng of each RNA in a final volume of 20 μl. For each sample, a triplicated blank was used. This cDNAs served as template in the PCR reaction carried out in 7900 HT Fast Real- Time PCR System using Power Sybrgreen PCR Master Mix 2× with 300 nM oligonucleotides concentration in a final volume of 10 μl. The results were acquired with SDS 2.3 software by Applied Biosystems and handled with EXCEL software by Microsoft. A total of seven genes were studied for each sample and their amount were related to one control gene, DDB0217951, whose expression is not affected by the treatments with the different strains.

## Authors' contributions

SC and JC performed the biological experiments and analyzed the data. GB, JS and AI designed and constructed the microarray, and provided informatics and analysis tools; GB contributed to the array experimental design, carried out the array experiments, and contributed to the analysis of results; RK was array project manager. JM contributed to the experimental design of the array and biological experiments. RE designed, coordinated the experiments and drafted the manuscript. All the authors read and approved the final manuscript.

## Supplementary Material

Additional file 1 Microsoft excel document containing all the results and genes contained in the microarray. Click here for file

Additional file 2 Microsoft excel document containing the array data for the genes that showed differences in both PAO1 and PA14. The genes were manually annotated and filtered for p < 0.05 and log2 ratio >0.5 or <-0.5. Table 2 was obtained from these data. Click here for file

Additional file 3 Microsoft excel document containing the array data and manual annotation of the genes that showed differential regulation between PAO1 and PA14. The genes were filtered for p < 0.05 and log2 ratio >0.5 or <-0.5. Table 3 was obtained from these data. Click here for file
